# Letter to the editor regarding - pleural effusion caused by *Trichinella spiralis* infection: two case reports

**DOI:** 10.1186/s12879-024-09163-w

**Published:** 2024-03-06

**Authors:** Jean Dupouy-Camet, Fabien Vaylet, Fabrizio Bruschi

**Affiliations:** 1 Faculté de Médecine, Paris Cité University, Paris, France; 2 Clinical Pneumology, Pôle de Santé du Plateau, Meudon La Foret, France; 3 Dipartimento di Ricerca Traslazionale, Scuola di Medicina, Pisa University, Pisa, Italy

**Keywords:** Pleural effusion, *Trichinella*, Trichinellosis, Antibodies

We read with interest the paper entitled “Pleural effusion caused by *Trichinella spiralis* infection: two case reports”, published by Pan and colleagues in a recent issue of your journal [1]. In brief, the authors described two cases of exudative pleurisy with a high level of mononuclear cells and a positive *Trichinella* serology. As a matter of fact, pleurisy is not a frequent occurrence during trichinellosis. According to Pawlowski [2], pleurisy can be observed during trichinellosis, either associated with migrating focal lung infiltrations (exudate), or because of hypoalbuminemia and/or circulatory complications (transudate). Gould [3] reports that “pleuritis is infrequent, generally dry, occurs mainly during the later weeks of infection, and is of short duration. A serous effusion may be a manifestation of congestive heart failure”. In the present case, the authors stated that due to a positive *Trichinella* serology and in the absence of other identified causes, these pleural effusions could be of parasite origin. However, the presence of *Trichinella* antibodies does not necessarily indicate an active infection, as the antibodies can persist in the blood for months or even years after the initial infection has been overcome [4]. Moreover, no clinical signs of acute trichinellosis (fever, myalgia, or facial edema) were reported. Rather, case 1 acquired trichinellosis from eating undercooked pork sausages 1.5 months before the occurrence of pleuritis and then showed gastrointestinal symptoms (diarrhea). These observations are not definitive proof of *Trichinella* infection. In addition, the patient had no muscular involvement, and it is well known that muscular pain usually lasts for several weeks after an infection with *Trichinella*. Pleural fluid cytology analysis of the two cases detected the presence of lymphocytes (not eosinophils), suggesting tuberculosis or malignancies, as mentioned by the authors. Nevertheless, case 1 displayed no obvious abnormality by either sonography (urological and cardiac), or positron emission tomography. Other possible causes of pleurisy were not investigated, such as viral infections or sarcoidosis, as well as connective tissue disorders [5, 6].

As mentioned by the authors, “the detection of parasite antibody IgG indicated *Trichinella spiralis* (+) by ELISA”. No information is provided about the method used (commercial or home-made test?), or the level of sensitivity and specificity. ELISA is a quantitative method for calculating antibody amounts. The sign + suggests only the presence of antibodies, but not the titer that if low might be compatible with an ancient infection. Several authors have reported on persistence of antibodies, explained by the long survival of *Trichinella* encapsulated larvae in muscles. Harms et al. [7] in a prospective controlled study of patients found that 38% of the 128 originally infected patients still had IgG antibodies to *Trichinella* ten years after acute infection. Kociecka et al. [8] identified antibodies in patients infected seven years before. In a study re-evaluating subjects 15 years after they were involved in a trichinellosis outbreak, Pinelli et al. [9] showed by different techniques (ELISA, western-blot) that antibodies were present in nearly all patients. Illic et al. [10] reported anti-*Trichinella* antibodies in 10 out of 12 patients who acquired trichinellosis between 13 and 18 years before. Therefore, the serological results must be evaluated in conjunction with the individual’s medical history, clinical symptoms, and other laboratory tests to establish a definitive diagnosis. Furthermore, it’s important to note that there are other conditions and infections that can cause a positive *Trichinella* antibody test, such as other parasitic infections, autoimmune disorders, or cross-reactivity with other antigens. These false-positive results can be eliminated by using more specific techniques, such as western-blot [11].

In the Discussion there are also probable typing or translation mistakes. When describing circulating antigens, the authors stated that these antigens were “produced by live insects”. And further, “after being treated with insect repellent (albendazole or mebendazole), pleural effusion was absorbed, so it was diagnosed as trichinosis”. *Trichinella* is not an insect, and albendazole and mebendazole are anthelminthic drugs and not insect repellents. The authors should also use throughout the text the word trichinellosis, instead of trichinosis, to designate the disease caused by *Trichinella*.

To resume, it’s difficult to confirm a link between pleurisy and *Trichinella*, since the search for alternative causes was not exhaustive combined with a lack of suggestive standard clinical and biological data consistent with active and/or recent trichinellosis. The favorable evolution could have been spontaneous if the pleural effusion had been caused by viruses. Albendazole and mebendazole could have enhanced this favorable evolution through their anti-inflammatory effects, as suggested by recent experimental studies in rodents [12, 13].

*This letter is dedicated to Fabien Vaylet who passed away after the initial submission of the manuscript. We also thank Gordon Langsley for the final language revision.*

## Data Availability

Not applicable.
